# Novel Androgen Receptor Inhibitors in Non-Metastatic, Castration-Resistant Prostate Cancer: A Systematic Review and Network Meta-Analysis

**DOI:** 10.3389/fonc.2021.733202

**Published:** 2021-10-15

**Authors:** Yelin Mulati, Yu Fan, Wei Yu, Qian Zhang, Zhisong He

**Affiliations:** ^1^ Department of Urology, Peking University First Hospital, Beijing, China; ^2^ Institute of Urology, Peking University, Beijing, China; ^3^ National Urological Cancer Center, Beijing, China; ^4^ Peking University Binhai Hospital, Tianjin, China

**Keywords:** non-metastatic castration-resistant prostate cancer (nmCRPC), hormonal therapies, overall survival (OS), adverse events, network meta-analysis

## Abstract

**Introduction:**

Enzalutamide, apalutamide, and darolutamide have all been approved by Food and Drug Administration to treat high-risk non-metastatic castration-resistant prostate cancer (nmCRPC) since 2018 based on interim results of several phase III clinical trials. Final analyses of long-term overall survival (OS) and adverse events (AEs) results of these trials have been successively published recently. To help clinical practice to precisely select optimal treatment for high-risk nmCRPC patients, we performed a network meta-analysis to indirectly compare the final long-term results among these medications.

**Methods:**

PubMed, EMBASE, and Cochrane Libraries were searched for phase III clinical trial that reports OS and AEs results in nmCRPC patients published before January 30, 2021. Primary outcome was OS; secondary outcomes were Time to first chemotherapy, Subsequent antineoplastic therapy rate, and AEs. Firstly, class-level effect was assessed as the second-generation androgen receptor antagonists (SGARAs) were regarded as one whole class compared with placebo through traditional meta-analysis by using Revman 5.4, then a Bayesian network meta-analysis was conducted to give indirect comparison among SGARAs by using R 3.5.3 software. Subgroup analysis of OS was only conducted in the certain subgroups which were available in all included studies.

**Results:**

Three eligible studies including 4,104 participants were finally selected. OS was significantly improved by the SGARAs as a class compared with placebo (HR, 0.74; 95% CI, 0.66–0.84). Darolutamide had the highest likelihood of providing best OS (p-score=0.802). SGARAs also significantly delayed the first time to chemotherapy (HR, 0.58; 95% CI, 0.50–0.66). Patients who received darolutamide experienced similar toxicity compared with placebo regarding AEs of grade 3 or higher (OR, 1.3; 95% CI, 1.0–1.7) and serious AEs (OR, 1.3; 95% CI, 0.99–1.6). When compared with darolutamide, enzalutamide caused significantly higher toxicity in terms of any AEs (OR, 2.3; 95% CI,1.5–3.7) and AEs of grade 3 or higher (OR, 1.6; 95% CI, 1.1–2.2), apalutamide caused significantly more AEs of grade 3 or higher (OR, 1.9; 95% CI, 1.4–2.7) and serious AEs (OR, 1.9; 95% CI, 1.3–2.8). Subgroup analysis showed that SGARAs as a group significantly improved OS in ECOG=1 population, although insignificant results were found in these patients from included studies.

**Conclusions:**

SGARAs combined with ADT significantly improved OS when compared with ADT alone in high-risk nmCRPC patients. Darolutamide may not only provide best OS but also have the most favorable safety profile among the included SGARAs in high-risk nmCRPC patients.

## Introduction

Non-metastatic castration-resistant prostate cancer (nmCRPC) is defined as prostate-specific antigen (PSA) progression and no evidence of distant metastases on conventional imaging in patients at castration levels of serum testosterone (1, 2). Observation plus continuous androgen-deprivation therapy (ADT) used to be the standard of care for all the nmCRPC patients (3, 4). Since 2018, second-generation androgen receptor antagonists (SGARAs), which include enzalutamide, apalutamide, and darolutamide, have all been successively approved by Food and Drug Administration (FDA) to treat high-risk nmCRPC (PSA doubling time <10 months), on the basis of the significant improvement of metastasis-free survival (MFS) in patients with high-risk nmCRPC receiving additional SGARA to ongoing ADT according to interim results of the three clinical phase III trials: PROSPER, SPARTAN, and ARAMIS studies (5–7). However, overall survival (OS) outcomes based on the interim data were immature, and potential benefits of OS provided by SGARAs were not significant. Recently, final analyses of the three trials reporting the long-term OS and adverse events (AEs) results have all been published (8–10). The three studies consistently showed significant improvement of overall survival accompanied with acceptable toxicity in patients who received SGARAs plus ADT compared with patients who received placebo plus ADT. However, the lack of head-to-head comparison of long-term OS and AEs results among the three SGARAs made it difficult to help the clinical practice precisely. As a result, we did a systematic review and meta-analysis to determine the efficacy and safety profile of SGARAs, then indirectly compared the latest long-term results of efficacy and safety among the SGARAs through Bayesian network meta-analysis to find out the optimal treatment for patients with high-risk nmCRPC.

## Materials and Methods

This study adhered to the recommendations of the preferred reporting items for systematic reviews and meta-analysis (PRISMA) extension statement for network meta-analysis (11, 12), and it was pre-registered in PROSPERO; the registration number is CRD42021231549.

### Study Selection

A systematic review was conducted through PubMed, EMBASE, and Cochrane Libraries on January 30, 2021. The searching strategy is specifically presented in **Supplement Material 1**.

All the references were imported into EndnoteX8 to be screened. Initial screening was conducted by two independent investigators based on title and abstract. Potential relevant studies were send to full-text review. A third author was consulted to resolve any disagreements between the two investigators.

We included phase III randomized clinical trials comparing OS and AE results of nmCRPC patients who received SGARA combining ADT with patients who received placebo plus ADT.

The following conditions were defined as exclusion criteria: (1) non-English studies; (2) absence of overall survival (OS) outcomes; (3) reviews, conference abstracts, protocols, comments.

### Data Extraction

A pre-designed Microsoft Excel table was used to extract general information and clinical characteristics from the studies finally included.

The primary outcome was overall survival (OS), and secondary outcomes were Time to first chemotherapy, Subsequent antineoplastic therapy rate, and Adverse events (AEs).

We extracted hazard ratio (HR)and 95% confidence intervals (CI) for primary and secondary endpoints if HR with 95% CI were available, or extracted number of events otherwise. Data extraction was performed by two independent authors. OS was defined as time from randomization to death of any cause. Time to first chemotherapy was defined as time from randomization to the first use of cytotoxic chemotherapy for prostate cancer. Subsequent antineoplastic therapy rate was defined as percentage of patients who received other new antineoplastic drugs. Adverse events (AEs) were defined and categorized according to the Common Terminology Criteria for Adverse Events (CTCAE) version 4.0.

### Statistical Analysis

Traditional meta-analysis of included studies was conducted initially to give an overall impression of SGARAs as one class compared with placebo. In this part, analyses were conducted using Review Manager5.3. Inverse variance technique was chosen for meta-analysis of HRs for efficacy outcomes, while the Mantel-Haenszel method was used for meta-analysis of binary variable data (e.g., AEs). Random effect model was applied in all analysis above. The risk of bias was assessed according to the Cochrane Collaboration’s tool for each included studies (13). Heterogeneity was assessed using I^2^ statistics during meta-analysis. I^2^ values greater than 25, 50, or 75% indicate low, moderate, or high heterogeneity, respectively. Secondly, we performed Bayesian network meta-analysis to compare the efficacy and safety of available SGARAs using Gemtic package in R 3.5.3 software.

Estimated differences in logHR and standard error were calculated based on published HRs and its 95% confidence intervals (CIs) to analyze efficacy outcomes (14). HR and 95% credible interval (Crl) were displayed as relative treatment effects. Estimated odds ratios (ORs) and 95% Crl were calculated for analysis of AEs using dichotomous data. Ranking probability and surface under the cumulative ranking curves (SUCRA) were synthesized to estimate the relative ranking of efficacy and safety of the candidate treatments. Random or fixed effect model was used where appropriate. Subgroup analysis of OS was conducted only in certain subgroups that were available in all the included studies.

## Results

A total of 927 publications were identified from initial database searching. **Supplementary Figure 1** shows the PRISMA flowchart of study selection procedure. After duplications removal, title and abstract screening, and full-text reviewing, three eligible studies including 4,104 participants were selected for final analysis (8–10). Baseline characteristics of the three included studies are summarized in **Table 1**. The three trials used enzalutamide+ADT, apalutamide+ADT, and darolutamide+ADT as intervention therapy, respectively.

**Table 1:** Baseline characteristics of included studies.

Characteristics		ARAMIS (2020)		PROSPER (2020)		SPARTAN (2020)	
Treatments		Darolutamide+ADT	Placebo+ADT	Enzalutamid+ADT	Placebo+ADT	Apalutamide+ADT	Placebo+ADT
Median age, y (range)		74 (48–95)	74 (50–92)	74 (50–95)	73 (53–92)	74 (48–94)	74 (52–97)
Median PSA ng/ml (range)		9.0 (0.3–853.3)	9.7 (1.5–885.2)	11.1 (0.8–1071.1)	10.2 (0.2–467.5)	7.78	7.96
Median PSADT, months		4.4	4.7	3.8	3.6	4.4	4.5
LN metastasis	YES	163 (17)	158 (29)	NR	NR	133 (16.5)	65 (16.2)
	NO	792 (83)	296 (71)	933 (100)	468 (100)	673 (83.5)	336 (83.8)
ECOG	0	650 (68)	391 (71)	747 (80)	382 (82)	623 (77)	311 (78)
	1	305 (32)	163 (29)	185 (20)	85 (18)	183 (23)	89 (22)
Bone target therapy	Yes	31 (3)	32 (6)	105 (11)	48 (10)	82 (10)	39 (10)
	No	924 (97)	522 (94)	828 (89)	420 (90)	724 (90)	362 (90)

Data presented as median (range) or n(%). PSA, prostate specific antigen; PSADT, PSA doubling time; LN, lymph node; NR, not reported; ECOG, Eastern Cooperative Oncology Group.

There was no difference of ranking results between the fixed effect model and random effect models with the former demonstrating a better fit in NMA. Risk of bias and quality assessment of included studies are shown in **Supplementary Figure 2**.

### Overall Survival

**Figure 1:**
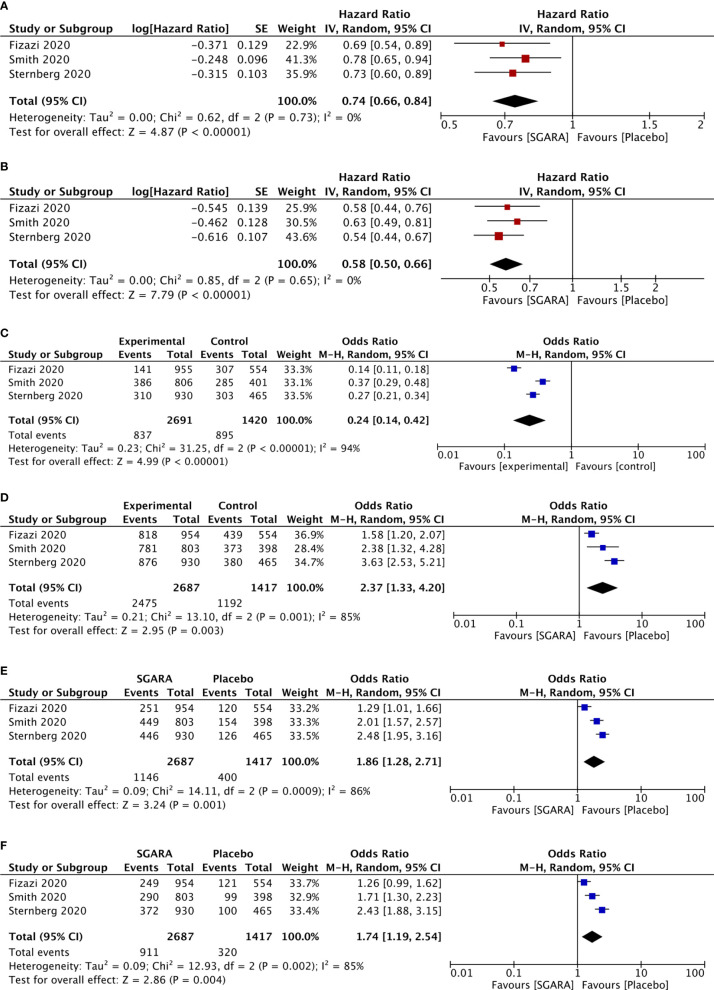
Meta-analysis results of included studies. **(A)** Overall survival. **(B)** Time to first chemotherapy. **(C)** The use of subsequent antineoplastic therapy. **(D)** Any adverse events. **(E)** Adverse events of grade 3 or higher. **(F)** Serious adverse events.

**Figure 2:**
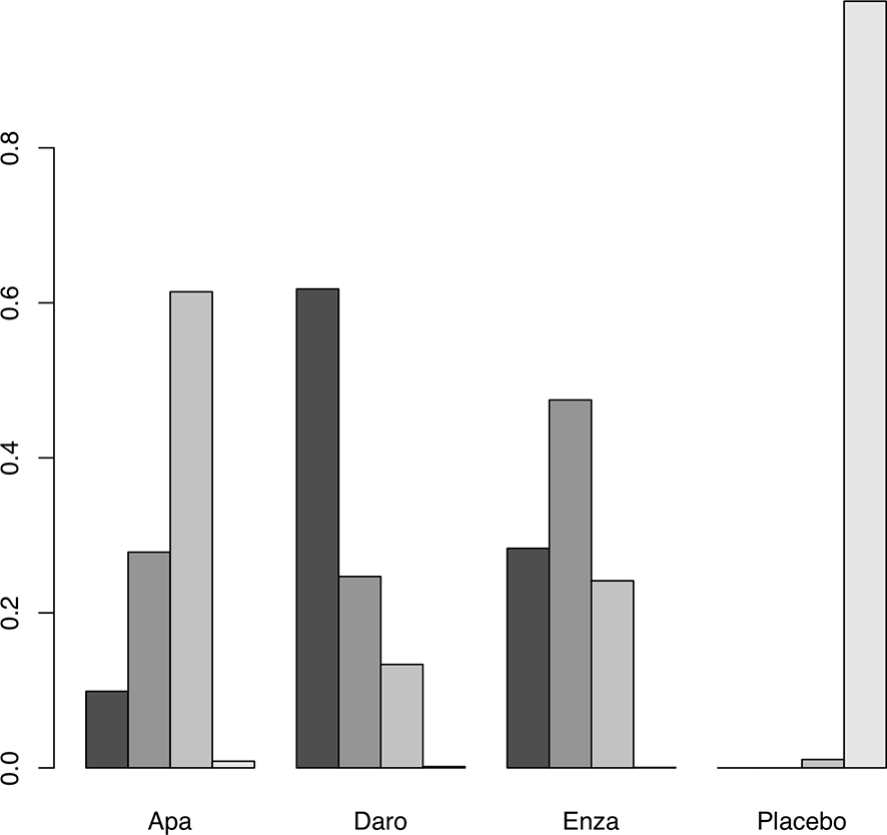
Surface under the cumulative ranking (SUCRA) plot of the treatments included. A darker color is proportional to a better performance in OS.

OS was significantly improved by SGARAs as one class compared with placebo in meta-analysis (HR, 0.74; 95% CI, 0.66–0.84), I^2 ^= 0% (see **Figure 1**). All the three agents significantly improved OS, respectively. There was no significant difference in OS among the SGARAs according to NMA. However, based on NMA results of OS ranking (see **Figure 2** and **Supplementary Figures 3**, **4**), darolutamide had the highest likelihood of providing the best OS (p-score=0.802), followed by enzalutamide and apalutamide (p-score=0.682 and 0.512, respectively).

### Subsequent Antineoplastic Therapy Rate

The use of subsequent antineoplastic therapy was significantly reduced by SGARAs compared with placebo (OR, 0.24; 95% CI, 0.14–0.42), I^2 ^= 94% (see **Figure 1**). In NMA (see **Supplementary Figures 5**, **6**), significantly more patients in enzalutamide group and apalutamide group used subsequent antineoplastic therapy compared with darolutamide group (OR, 1.9, 2.7; 95% CI, 1.4–2.7, 1.9–3.8, respectively).

### Time to First Use of Chemotherapy

SGARAs significantly delayed the time to first use of chemotherapy compared with placebo (HR, 0.58; 95% CI, 0.50–0.66), I^2 ^= 0% (see **Figure 1**). No significant difference was found among SGARAs regarding the time to first use of chemotherapy in NMA.

### Adverse Events

**Figure 3:**
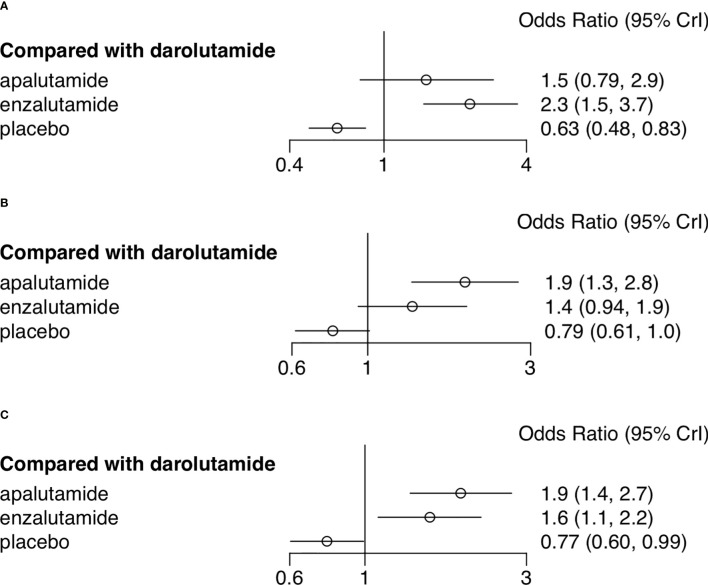
Network meta-analysis forest plot of adverse events of treatments compared with darolutamide. **(A)** Any adverse events. **(B)** Adverse events of grade 3 or higher. **(C)** Serious adverse events.

AEs were assessed through multiple endpoints, included any AEs, AEs of grade 3 or higher, and serious AEs (SAE). SGARAs as a class were associated with significantly higher toxicity no matter which endpoint was assessed (see **Figure 1**). Darolutamide experienced similar toxicity compared with placebo according to AEs of grade 3 or higher (OR, 1.3; 95% CI, 1.0–1.7), SAEs (OR, 1.3; 95% CI, 0.99–1.6) (see **Figure 2**). Enzalutamide and apalutamide caused significantly higher toxicity than darolutamide according to AEs of grade 3 or higher (OR, 1.6, 1.9; 95% CI, 1.1–2.2, 1.4–2.7). Enzalutamide was associated with significantly higher toxicity than darolutamide according to any AEs (OR, 2.3; 95% CI, 1.5–3.7). Apalutamide was associated with significantly higher toxicity than darolutamide according to serious AEs (OR, 1.9; 95% CI, 1.3–2.8) (see **Figure 3**).

### Subgroup Analysis

**Figure 4:**
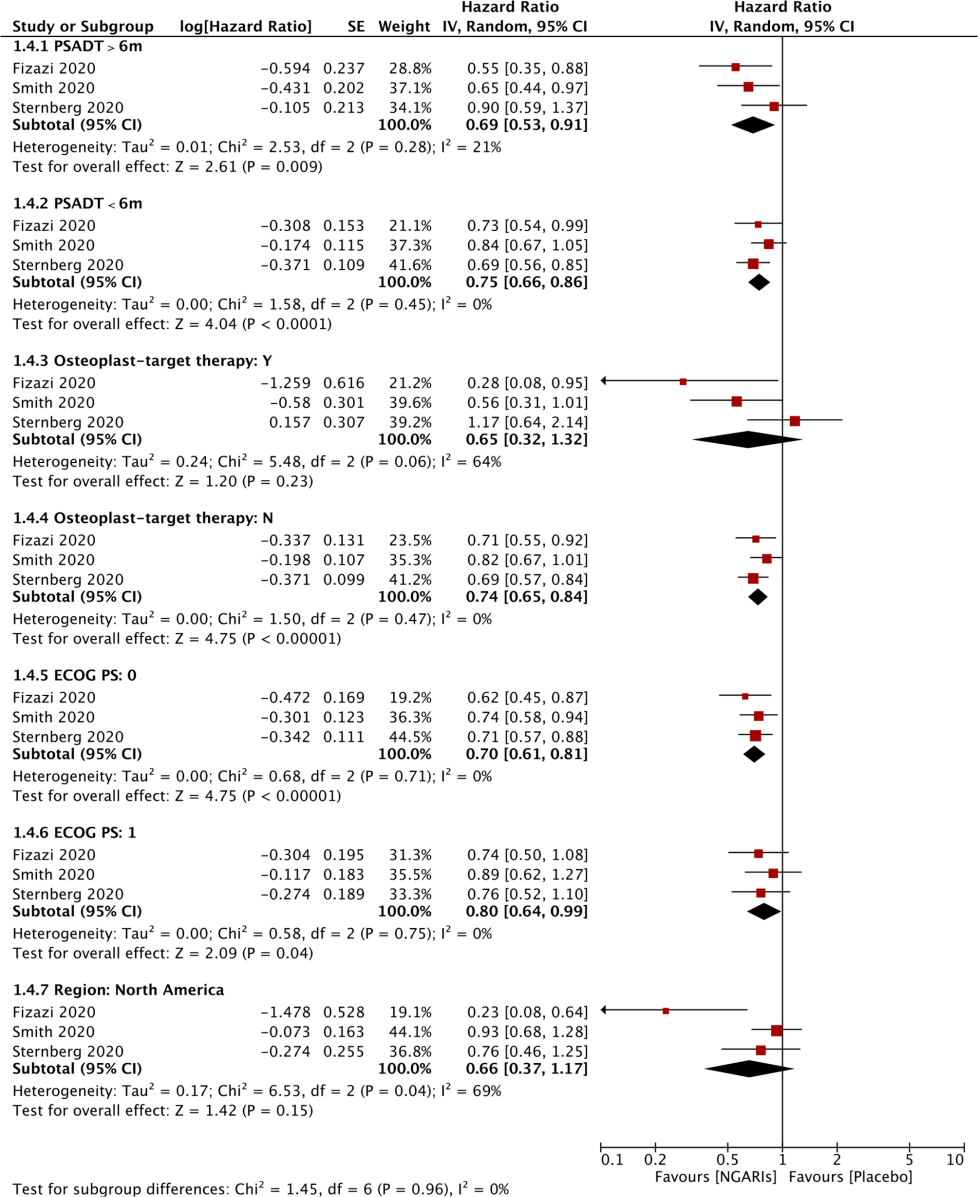
Subgroup analysis of overall survival.

Subgroup analysis was conducted only in certain subgroups that were available in all three studies, including PSA doubling time, baseline osteoplast-targeting therapy, ECOG performance-status, and region (North America). OS was significantly improved in patients who received SGARAs compared with placebo across all these subgroups, except patients with baseline osteoplast-targeting therapy and patients in North America. Although the improvement was not significant in each included studies respectively in ECOG 1 patients, it became significantly improving OS when SGARAs were regarded as a class compared with placebo in ECOG 1 patients (HR, 0.80, 95% CI, 0.64–0.99) (see **Figure 4**). In NMA analysis, OS in patients who received osteoplast-targeting therapy was significantly inferior in patients who received enzalutamide than patients who received darolutamide (**Supplementary Figure 7**). In the region of North America, darolutamide improved OS significantly compared with apalutamide and enzalutamide (**Supplementary Figure 8**).

## Discussion

The landscape of treatment for nmCRPC patients has evolved (15).

FDA has approved enzalutamide, apalutamide, and darolutamide to treat nmCRPC. To guide clinical practice, several NMA studies have been conducted and demonstrated that apalutamide and enzalutamide may provide better MFS than darolutamide, and apalutamide may have the best MFS based on interim results of the three trials (16–19).

According to our study based on the recently published long-term results of OS and AEs, we found that darolutamide not only showed a potential advantage of OS compared with enzalutamide and apalutamide, but also showed best tolerance in terms of AEs in patients with high-risk nmCRPC. It is necessary to point out that patients with known central nervous system malignancies were excluded in PROSPER and SPARTAN, while they were included in the ARAMIS study.

The advantages of darolutamide may be due to the unique molecular structure distinct from enzalutamide and apalutamide, as it gives darolutamide a higher androgen receptor binding affinity and negligible penetration of blood-brain barrier according to the preclinical study (20, 21). In addition, darolutamide can also block the mutant ARs arising in response to ADT, which conferred resistance to enzalutamide and apalutamide (22).

The three trials allowed patients in the placebo group to cross over to receive open-label treatment drug (SGARA) after unblinding treatment assignments, and all these crossed-over patients were still included into placebo group for final OS analysis. Therefore, the more the patients crossed over from placebo group to treatment group, the less significant the potential improvement for OS of treatment drug would be. The crossed over rates differed among the studies. There were 170 of 544 patients (31%), 76 of 401 patients (19%), 87 of 465 patients (19%) in placebo group that crossed over to receive open-label treatment regimen in ARAMIS, SPARTAN, and PROSPER study, respectively. On the contrary, the more patients received the subsequent life-prolonging therapy, the effect of improving OS of treatment drug was more likely to be overestimated. Significantly more patients in enzalutamide group and apalutamide group received subsequent life-prolonging therapy than darolutamide group (OR, 1.9, 2.7; 95% CI, 1.4–2.7, 1.9–3.8). As a result, though darolutamide has had the highest likelihood of providing best OS, we still assumed that the advantage was underestimated.

Improving OS of cancer patients is the ultimate goal of antineoplasm drug. Our study, applying the latest long-term OS outcomes, showed darolutamide may provide best OS among the three included SGARAs, which was different from the previous NMA results using early OS data (16–18). Previous NMA also showed significantly better MFS in patients who received apalutamide or enzalutamide compared with patients who received darolutamide. However, we found that different censoring rules for MFS analysis were applied in ARAMIS study compared with SPARTAN and PROSPER studies. In PROSPER and SPARTAN trials, patients who were randomly assigned to the study and later found to have had baseline metastatic disease at central review would be left censored for time-to-event analysis (which happened 16 weeks after randomization) (6). In contrast, ARAMIS trial right censored these patients at the date of randomization (5). This difference brought in heterogeneity and may underestimate the MFS of darolutamide.

According to our subgroup analysis, insignificant OS outcome was found in the North American population with SGARAs compared with placebo (HR, 0.66; 95% CI, 0.37–1.17). European and Asian subgroup analysis failed to be performed due to different cutoff levels across the three studies.

Comparable efficacy and similar safety outcomes were found between the Japanese subgroup population and globally overall population in both ARAMIS and SPARTAN studies (23, 24). However, significantly more skin rash cases were reported in the Japanese subgroup population compared with the overall population in the SPARTAN study (56 *vs* 23.8%) (24), and skin rash was documented as the most common reason for treatment discontinuation (7).

In the TITAN study, patients with mHSPC who received apalutamide in the Japanese subgroup population had relatively inferior primary efficacy outcomes than the overall population (25). These results suggested that SGARA may also have distinct efficacy or AE outcomes in the Asian population from the overall population. More studies are expected to investigate the outcomes of SGARAs in Asian population, especially studies in Chinese population.

This is a valuable network meta-analysis using the latest long-term outcomes of OS and AEs to compare the efficacy and safety of SGARAs in patients with high-risk nmCRPC, which may provide valuable information to both urologists and nmCRPC patients. But there were still some limitations that should be noticed. First, though NMA was conducted to outline a rough picture of indirect comparison of efficacy and safety among SGARAs, direct head-to-head comparisons among SGARAs were still lacking, to combine with indirect outcomes for a more convincing pooled outcome; heterogeneity also failed to be evaluated through NMA. Second, subgroup analysis of OS was failed to be performed in many subgroups due to lack of data and different cutoff levels across the included studies, so further exploration of overall survival in different subgroup populations cannot be carried out. Subgroup classification in studies needs to be standardized and unified in the future. In addition, patients’ baseline characteristics may have significant difference among included studies, which may affect the comparability of outcomes from the different studies. For example, patients with previous seizure or conditions predisposing to seizure were excluded in PROSPER and SPARTAN trials (6, 7), while included in ARAMIS trial (5). And N1 patients were included in ARAMIS and SPARTAN trials, while PROSPER study only included N0 patients.

## Conclusion

SGARAs combined with ADT significantly improved OS when compared with ADT alone in nmCRPC patients. Darolutamide may provide potentially best OS, and at the same time, it appeared to have the most favorable safety profile among the included SGARAs in high-risk nmCRPC patients. Direct head-to-head comparison among SGARAs is required to confirm these findings.

## Data Availability Statement

The raw data supporting the conclusions of this article will be made available by the authors, without undue reservation.

## Author Contributions

All authors listed have made a substantial, direct, and intellectual contribution to the work and approved it for publication.

## Funding

This work was supported by the National key research and development program of China (Grant No. 2017YFC0908003, 2017YFC0110900), National Natural Science Foundation of China(Grant No. 82072826) and Special Project of “Group Medical Assistance Project” of Tibet Autonomous Region Health Committee (Grant No. XZ2019ZR-ZY16(Z)).

## Conflict of Interest

The authors declare that the research was conducted in the absence of any commercial or financial relationships that could be construed as a potential conflict of interest.

## Publisher’s Note

All claims expressed in this article are solely those of the authors and do not necessarily represent those of their affiliated organizations, or those of the publisher, the editors and the reviewers. Any product that may be evaluated in this article, or claim that may be made by its manufacturer, is not guaranteed or endorsed by the publisher.

## Supplementary Material

Supplementary Figure 1 Flowchart of studies selection process.

Supplementary Figure 2 Risk of bias assessment of included studies.

Supplementary Figure 3 Network meta-analysis OS p-score plot of treatments. A higher p-score represent a higher possibility to have better OS.

Supplementary Figure 4 Network meta-analysis forest plot of overall survival of treatments compared with darolutamide.

Supplementary Figure 5 Network meta-analysis forest plot of the use of subsequent antineoplastic therapy, other treatments compared with darolutamide.

Supplementary Figure 6 Surface under the cumulative ranking (SUCRA) plot of the treatments included. A darker color is proportional to a less use of subsequent antineoplastic therapy.

Supplementary Figure 7 Network meta-analysis forest plot of subgroup patients who received osteoplast-targeting therapy, other treatments compared with darolutamide.

Supplementary Figure 8 Network meta-analysis forest plot of subgroup patients in the region of North America, other treatments compared with darolutamide.

Supplement Material 1 (“non metasta*”[Title/Abstract] OR (“non”[All Fields] AND “metasta*”[All Fields]) OR “non metasta*”[Title/Abstract] OR “local*”[Title/Abstract]) AND “randomized controlled trial”[Publication Type] AND ((“prostatic neoplasms, castration resistant/drug therapy”[MeSH Terms] OR ((“androgen”[Title/Abstract] OR “castration”[Title/Abstract] OR “hormone”[Title/Abstract]) AND (“independent”[Title/Abstract] OR “insensitive”[Title/Abstract] OR “resistant”[Title/Abstract] OR “refractory”[Title/Abstract]) AND “prostat*”[Title/Abstract] AND (“cancer*”[Title/Abstract] OR “neoplasm*”[Title/Abstract] OR “tumor*”[Title/Abstract]))) AND (“abiraterone”[Title/Abstract] OR “apalutamide”[Title/Abstract] OR “darolutamide”[Title/Abstract] OR “enzalutamide”[Title/Abstract] OR (“androgen receptor antagonists”[Pharmacological Action] OR “androgen receptor antagonists”[MeSH Terms] OR (“androgen”[All Fields] AND “receptor”[All Fields] AND “antagonists”[All Fields]) OR “androgen receptor antagonists”[All Fields]) OR (“antineoplastic agents”[Pharmacological Action] OR “antineoplastic agents”[MeSH Terms] OR (“antineoplastic”[All Fields] AND “agents”[All Fields]) OR “antineoplastic agents”[All Fields])) AND “randomized controlled trial”[Publication Type]).
