# Supplementary material for: Novel Androgen Receptor Inhibitors in Non-Metastatic, Castration-Resistant Prostate Cancer: A Systematic Review and Network Meta-Analysis
Source: Front Oncol. 2021 Oct 15;11:733202. doi: 10.3389/fonc.2021.733202 (PMC8555656; doi:10.3389/fonc.2021.733202)
Supplement: Supplementary Figure 1 — Flowchart of studies selection process. [file Presentation_1.pdf]

Supplementary Materials

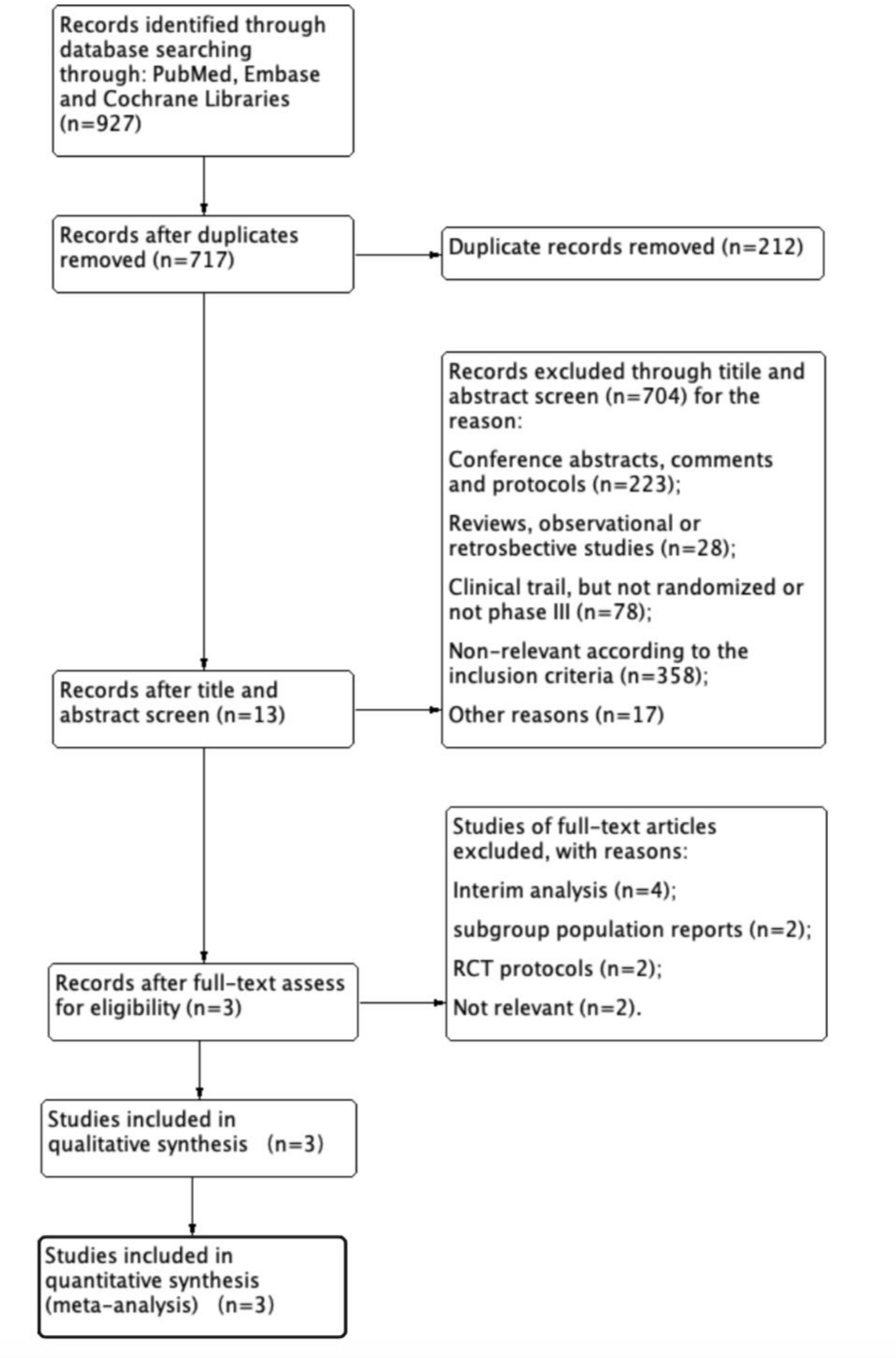

Supplementary Fig 1 Flowchart of studies selection process.

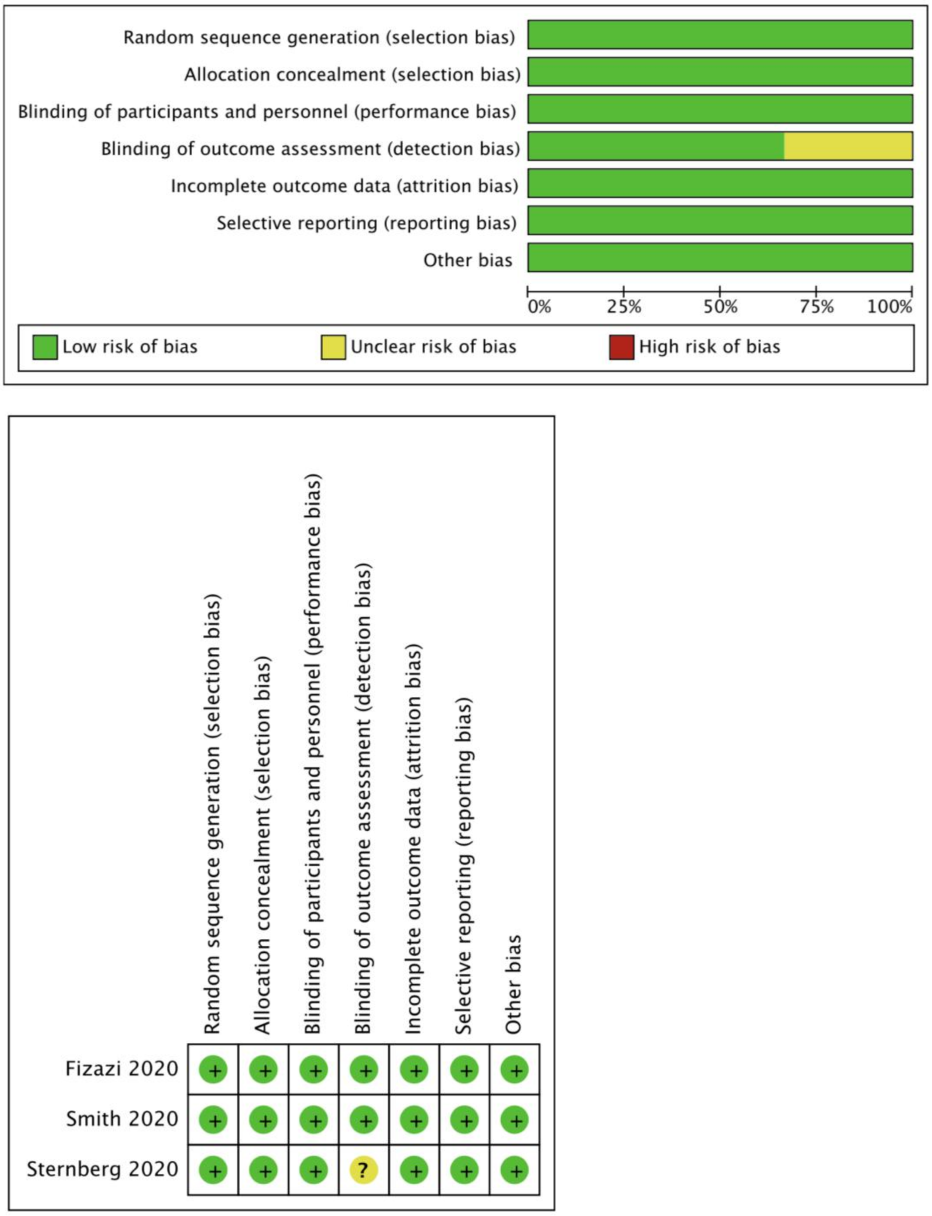

Supplementary Fig 2 Risk of bias assessment of included studies.

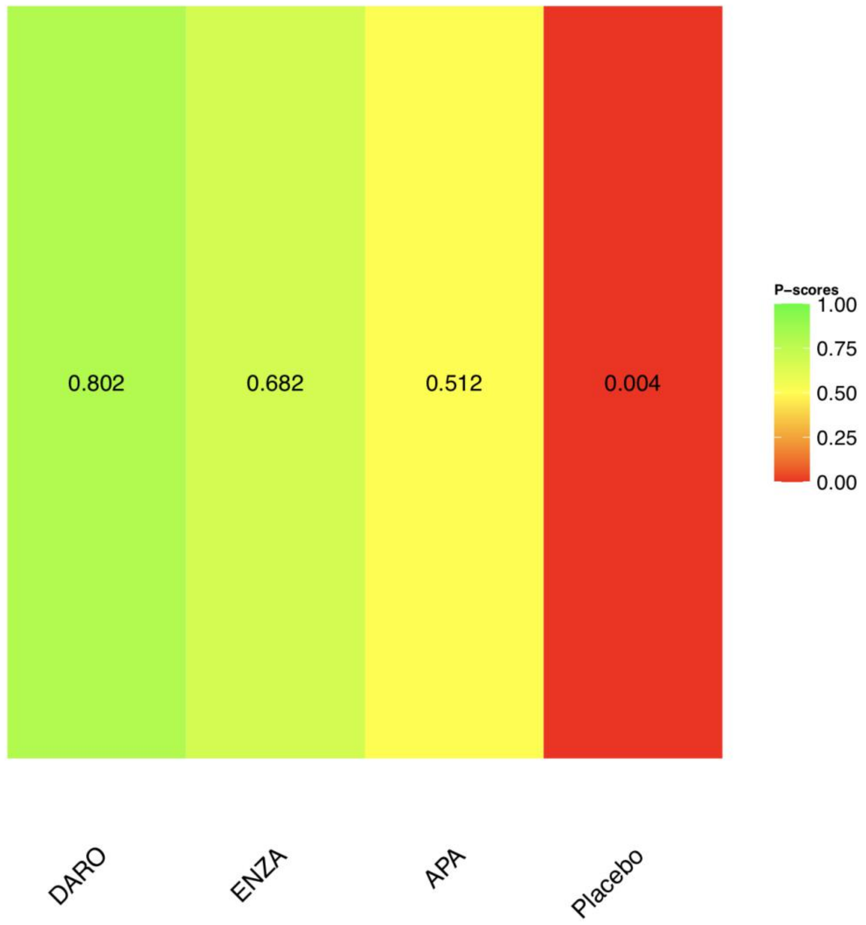

Supplementary Fig 3 Network meta-analysis OS p-score plot of treatments. A higher p-score represent a higher possibility to have better OS.

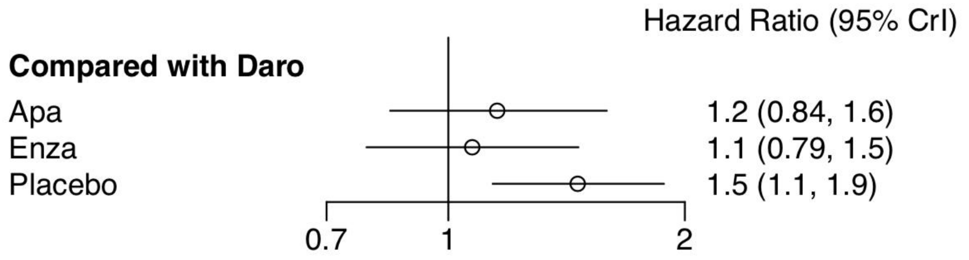

Supplementary Fig 4 Network meta-analysis forest plot of overall survival of treatments compared with darolutamide

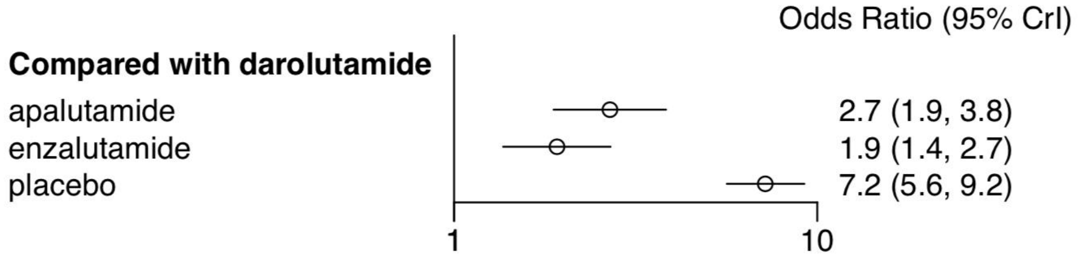

Supplementary Fig 5 Network meta-analysis forest plot of the use of subsequent antineoplastic therapy, other treatments compared with darolutamide.

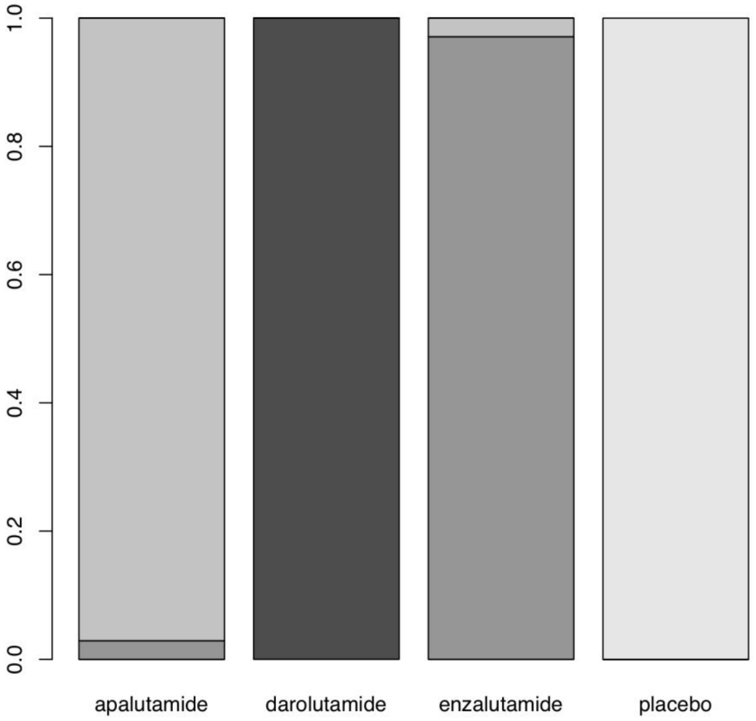

Supplementary Fig 6 Surface under the cumulative ranking (SUCRA) plot of the treatments included. A darker color is proportional to a less use of subsequent antineoplastic therapy.

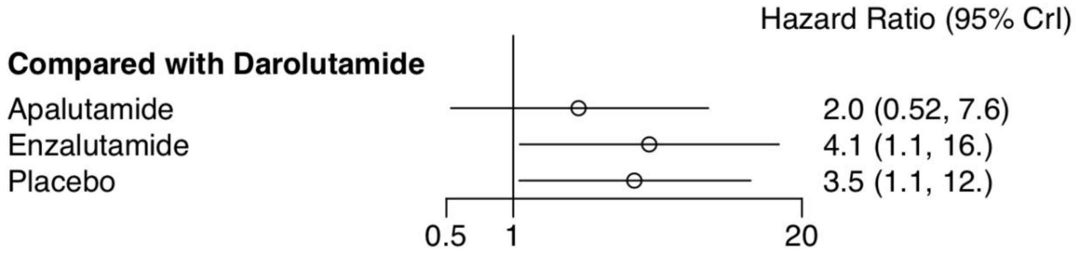

Supplementary Fig 7 Network meta-analysis forest plot of subgroup patients who received osteoplast-targeting therapy, other treatments compared with darolutamide.

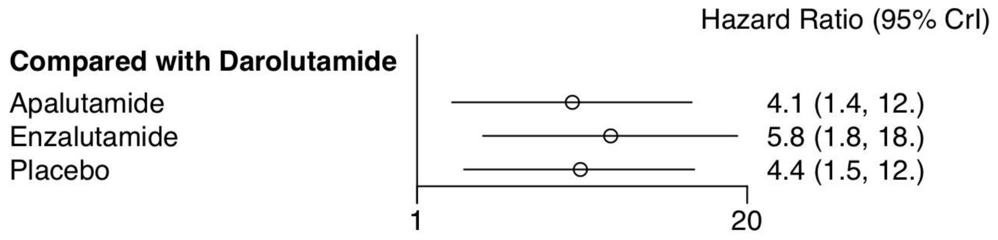

Supplementary Fig 8 Network meta-analysis forest plot of subgroup patients in the region of North America, other treatments compared with darolutamide.

Supplement material 1

("non metasta*"[Title/Abstract] OR ("non"[All Fields] AND "metasta*"[All Fields]) OR "non metasta*"[Title/Abstract] OR "local*"[Title/Abstract]) AND "randomized controlled trial"[Publication Type] AND (("prostatic neoplasms, castration resistant/drug therapy"[MeSH Terms] OR (("androgen"[Title/Abstract] OR "castration"[Title/Abstract] OR "hormone"[Title/Abstract]) AND ("independent"[Title/Abstract] OR "insensitive"[Title/Abstract] OR "resistant"[Title/Abstract] OR "refractory"[Title/Abstract]) AND "prostat*"[Title/Abstract] AND ("cancer*"[Title/Abstract] OR "neoplasm*"[Title/Abstract] OR "tumor*"[Title/Abstract]))) AND ("abiraterone"[Title/Abstract] OR "apalutamide"[Title/Abstract] OR "darolutamide"[Title/Abstract] OR "enzalutamide"[Title/Abstract] OR ("androgen receptor antagonists"[Pharmacological Action] OR "androgen receptor antagonists"[MeSH Terms] OR ("androgen"[All Fields] AND "receptor"[All Fields] AND "antagonists"[All Fields]) OR "androgen receptor antagonists"[All Fields]) OR ("antineoplastic agents"[Pharmacological Action] OR "antineoplastic agents"[MeSH Terms] OR ("antineoplastic"[All Fields] AND "agents"[All Fields]) OR "antineoplastic agents"[All Fields])) AND "randomized controlled trial"[Publication Type])
